# Role of phospholipase C in banana in response to anthracnose infection

**DOI:** 10.1002/fsn3.1388

**Published:** 2020-01-10

**Authors:** Liang Shuai, Li Li, Jian Sun, Lingyan Liao, Zhenhua Duan, Changbao Li, Xuemei He

**Affiliations:** ^1^ Guangxi Crop Genetic Improvement and Biotechnology Key Laboratory Guangxi Academy of Agricultural Sciences Nanning China; ^2^ College of Food and Biological Engineering/Institute of Food Science and Engineering Technology Hezhou University Hezhou Guangxi China; ^3^ Guangxi Key Laboratory of Fruits and Vegetables Storage‐processing Technology Guangxi Academy of Agricultural Sciences Nanning China; ^4^ Agro‐food Science and Technology Research Institute Guangxi Academy of Agricultural Sciences Nanning China

**Keywords:** anthracnose resistance, banana, phospholipase C

## Abstract

Phospholipase C (PLC) plays an important role in plant immunity, and anthracnose caused by the *Colletotrichum* species is a common postharvest disease of the banana fruit. This study aims to evaluate the role of PLC in anthrax resistance in banana. The experimental group of banana samples was treated with a banana anthracnose conidia suspension, and the control group was treated with distilled water. After inoculation, the groups were sprayed with ethephon, and indicators, such as hardness and conductivity changes; PLC activity, 1,2‐diacylglycerol (DAG) and phosphatidic acid (PA)content; and *MaPLC‐1*and *MaPLC‐2* expression levels, were assessed at 0, 3, 6, 9, 12, and 15 days. Moreover, the expression levels of *MaPLC‐1* and *MaPLC‐2* were detected in various tissues. The hardness of banana fruits in the experimental group decreased faster than that in the control group. Furthermore, the conductivity was higher in the experimental group than in the control group. Regarding PLC activity, DAG, and PA content, bananas in the experimental group showed higher activities than those in the control group. Moreover, relatively higher expression of PLC mRNA was detected in anthracnose‐inoculated tissues. The evaluation of *MaPLC‐1* and *MaPLC‐2* expression levels showed that the mature peel had the highest *MaPLC‐1* expression level. However, the *MaPLC‐2* gene was expressed at relatively low levels in the fruit and at relatively high levels in the flower organs. PLC might play a role in fruit ripening in response to anthracnose resistance.

## Graphical abstract

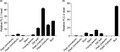

## 1 INTRODUCTION

In the tropics and subtropics, banana is widely accepted as a major commercial fruit crop that plays a key role in the economy of developing countries (Chabi et al., 2018; Li, Limwachiranon, Li, Du, & Luo, 2016). However, because of their highly perishable nature, banana fruits usually have a short shelf life after harvest under tropical conditions (Luo et al.; Wang et al.). Banana anthracnose, also known as black rot, is a fungal disease caused by *Calletotrichum musae* that widely occurs in all banana areas, mainly causing fruit damage (Intan Sakinah, Suzianti, & Latiffah, 2014), which is a common postharvest disease of banana fruits (Abayasekara, Adikaram, Wanigasekara, & Bandara, 2013). Postharvest disease significantly reduces the commercial value of banana fruits (Campuzano, Rosell, & Cornejo, 2018; Wang et al., 2013). To date, the control of banana anthracnose mainly includes resistant variety selection; clearing and destroying the source of the disease in time; and early bagging, chemical control, and fruit harvest on sunny days (Intan Sakinah et al., 2014; Sridhar & Sohi, 1974). However, the incidence of this disease remains high. With societal development, consumers’ expectation of fruit quality has become an ongoing challenge. Flesh bruising and body rots (principally anthracnose) are the main postharvest quality defects of banana fruits. Therefore, new resistant varieties are urgently needed.

Many researchers have focused on exploring the mechanism associated with durable or partial resistance to anthracnose in plants, and genetic mapping and candidate gene analysis have been established for the identification of resistance genes, such as resistance gene analogs derived from different R‐gene motifs and calcium/calmodulin‐dependent signaling pathways (Ahn, Kim, Choi, & Lee, 2003; Lopez et al., 2003; Neary et al., 1999). Accumulating studies have indicated that phospholipid‐based signal transduction is widely used by the plant cells to relay the perception of extracellular signals (Nakamura, 2017; Poulsen et al., 2015). Several phospholipid‐hydrolyzing enzymes are activated and established as an appropriate defense response with perception of the invading microbe, which is found in various organisms, such as bacteria, yeast, plants, animals, and viruses (Boller & Felix, 2009; Munnik, 2001). Phospholipase C (PLC) is an enzyme that catalyzes the hydrolysis of phosphatidylinositol 4,5‐bisphosphate to inositol 1,4,5‐trisphosphate (IP3) and diacylglycerol (DAG), which are involved in the activation of more than 100 different cell surface receptors (Rebecchi & Pentyala, 2000). In animals, PLC functions as a major membrane phospholipid‐hydrolyzing enzyme and generates signaling messengers, such as DAG and IP3. The roles of PLC in various animal diseases, such as coronary heart disease and Alzheimer's disease, have been widely researched (Albasanz, Dalfo, Ferrer, & Martin, 2005; Ballantyne et al., 2005, 2004). In plants, phosphorylated forms of PLC, such as phosphatidic acid (PA) and inositol hexakisphosphate, are thought to regulate various cellular processes. PLC is ubiquitous in plant tissues and is mainly involved in cell growth and differentiation, hormone signal transduction, response to biotic and abiotic stresses, and the regulation of polar growth. PLCs in plants are classified into phosphoinositide‐specific PLC (PI‐PLC), nonspecific PLC, and glycosyl‐phosphatidylinositol PLC (Khan, Alexander, Ajazuddin, Saraf, & Saraf, 2013; Wissing & Behrbohm, 1993). The PLC enzyme mainly hydrolyzes phospholipids to generate DAG and IP3. Traditionally, PLC activity is related to various factors, such as Ca^2+^ concentration, phospholipid substrate, post‐translational modifications, and interacting proteins. For example, previous studies have shown that the signaling pathway of PI‐PLC is involved in the perception of pathogen‐derived elicitors (Moser et al., 1997; Tsai & Chung, 2014). Vossen and colleagues have demonstrated that there is a differential requirement of PLC isoforms for plant immune responses and that PLC‐6 may be a more general component of resistance protein signaling (Vossen et al., 2010). Therefore, PLC might be important in banana anthracnose resistance.

Therefore, to evaluate the role of PLC in banana anthracnoseresistance, a banana anthracnose model was designed in our study. The characteristics of fruit hardness and electrical conductivity are widely used as indexes to evaluate fruit quality. Several indicators, such as changes in hardness; banana fruit membrane permeability; PLC activity, DAG, and PA content; as well as the expression levels of *MaPLC‐1* and *MaPLC‐2*, were assessed.

## 2 MATERIALS AND METHODS

### 2.1 Plant materials

Banana (*Musa acuminata* L.) tissues, including the floral bud, flower, green and senescent leaves, pseudostem, stem, developing and mature fruits, and fruits at different postharvest stages were collected from a commercial orchard at Nanning, Guangxi province during July 2018 and were immediately transported to the laboratory. Banana fruits with similar size and the same maturity period, without infection and physical damage, were chosen and randomly subdivided into experimental and control groups (three fruits in each group).

### 2.2 Fungal strain culturing and sample processing

The fungus was isolated from banana fruits with the symptoms of typical anthracnose via tissue separation. Water droplets on the surface were dried using sterile absorbent paper. Then, the diseased peel was cut into small pieces of 5 mm each, and these small pieces were soaked in 70% alcohol solution for 3–5 s. The pieces were then placed into a 0.1% mercury solution for 30–60 s. After washing 3–4 times with sterilized water, the pieces were placed onto potato agar medium (PDA) plates with five pieces of diseased tissue on each plate, at 25°C and 95% relative humidity. The status of colony formation was observed every day until the characteristic colonies grew around the diseased tissue block, and the diameter of the colony was 20–30 mm. Finally, culture purification was performed by inoculating the edge of the colony on a PDA plate for 6 days at 28°C.

Banana fruits in the experimental group were treated with a banana anthracnose conidia suspension (10^6^ spores/ml) via artificial spraying, whereas the control group was treated with distilled water. After the treatment, the banana piece was packed in a 0.03‐mm‐thick polyethylene film bag and stored at 20°C to observe the occurrence of anthracnose. Physiological indicators were detected before inoculation, and samples were collected at 0, 3, 6, 9, 12, and 15 days after inoculation. Six fruits were sampled each time.

### 2.3 Hardness and conductivity measurement

Two grams of banana peel was used to measure conductivity. The peel was rinsed three times with distilled water, dried using filter paper, and placed into a 50 ml centrifuge tube containing 20 ml of distilled water. After 1 hr of shaking, the conductivity was measured using a conductivity meter. Conductivity was further measured at the time of the peel cooling to room temperature after boiling for 20 min. Relative conductivity was defined as the ratio of the two conductivities, where the relative conductivity represents cell membrane permeability. The experiment was repeated three times.

The GY‐4 fruit hardness tester (AMETEK Test & Calibration Instruments) was used to measure the hardness of the fruit. The bananas were peeled, and the fruits were cut from the middle. Three fruits were randomly taken each time. Two points were measured for each cut surface, and the average of the six values was defined as the hardness of the fruit.

### 2.4 PLC enzymatic, DAG, and PA content assays

Bananas (5 g) were grinded for 10 min with 3 ml of 0.05 M sodium phosphate buffer (pH 7.8). Then, 10% of the homogenate was centrifuged for 15 min at 1500 *g*/min, and the supernatant was collected as an enzyme solution for enzyme‐linked immunoreactivity detection.

Both the sample and blank control wells were prepared in the experiment. First, 50 μL of the sample dilution (1:5) was added to the sample well on the enzyme‐labeled plate. Next, 100 μL of enzyme standard reagent was added to the sample wells. After sealing the plate with a sealing film, the plate was incubated at 37°C for 60 min. Thereafter, the sealing film was carefully removed, and the liquid was discarded and dried. The washing solution was added to each well and incubated for 30 s and then discarded. The procedure was repeated five times. The sample was finally subjected to a color reaction at 37°C for 15 min in the dark after adding color reagent. The reaction was stopped by adding 50 μl of the stop solution to each well. After 15 min, the OD value was measured at 450 nm.

### 2.5 RNA extraction

Total RNA was extracted from the samples using the RNA Prep Pure Plant Kit (Tiangen Co.) following the manufacturer's instructions. After extraction, total RNA concentrations were measured at 260 and 280 nm using a NanoDrop1000 spectrophotometer (NanoDrop Technologies). The RNA was qualified when the OD260/OD280 ratio ranged between 1.7 and 2.1 and the concentration was >10 ng/μl.

### 2.6 Real‐time quantitative PCR (QPCR) analysis

Reverse transcription (RT) was performed using the HiScript II Q RT SuperMix for qPCR (Vazyme Biotech Co., Ltd). In brief, 1ng of RNA, 2 μl of 5× HiScript II qRT SuperMix II, and RNase‐free water were mixed to a final volume of 10 μl. cDNA was obtained after incubating the samples at 50°C for 15 min and then at 85°C for 5 s. The mRNA expression levels of *MaPLC‐1* and *MaPLC‐2* were detected using RT‐PCR analysis. The expression levels of *MaPLC‐1* and *MaPLC‐2* in different tissues samples, including the immature peel and flesh, root, stem, young and old leaves, mature peel and flesh, and the bud, were further detected. Primers for *MaPLC‐1* (PLC‐1‐F: 5‐TTGAGCACTCTTCCGTTCCA‐3; PLC‐1‐R: 5‐ATCAGTTCTTGGCGTCTTCCT‐3) and *MaPLC‐2* (PLC‐2‐F: 5‐GGGATGAATGGGCTGGAACT‐3; PLC‐2‐R: 5‐GACAATTTCCCTTAGCAG‐3) were employed for RT‐PCR. The expression levels of genes were calculated using the standard $2^{-\Delta \Delta C_{T}}$ method, with *CAC (CAC‐F: CTCCTATGTTGCTCGCTTATG; CAC‐R: GGCTACTACTTCGGTTCTTTC)* as internal controls. The real‐time PCR conditions were as follows: preincubation at 95°C for 5 min followed by 45 cycles at 95°C for 15 s, 57°C for 15 s, and 72°C for 20 s.

### 2.7 Statistical analysis

All experiments were conducted in triplicate, and the results were presented as mean ± standard error of three replicates. Independent Student's *t* test was used to compare the difference between the two groups. All statistical analyses were performed using SPSS 24.0 statistical software (IBM, Armonk, New York). A *p*‐value < .05 was defined as statistically significant.

## 3 RESULTS

### 3.1 Hardness and conductivity evaluation

**Figure 1:**
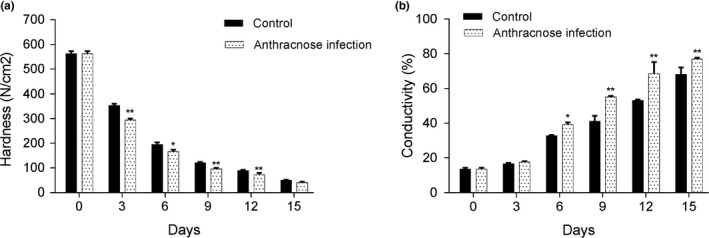
Comparison of the hardness (a) and conductivity (b) of banana in the anthracnose infection and control groups. The experiment was repeated three times. *p < .05 and **p < .01 indicate significant difference with control group

The hardness and conductivity of the banana anthracnose infection and control groups were shown in Figure 1. From the third day, the hardness of the banana samples in the infection group was significantly lower than that of the control group. During the first 15 days, the hardness of banana samples in the anthracnose infection group decreased faster than that of the control group (Figure 1a). Notably, after the third day, the anthracnose infection group showed higher conductivity than the control group. Anthracnose infection reduced the hardness and increased the conductivity of bananas, indicating that anthracnose infection destroyed the cell membrane structure of the bananas (Figure 1b).

### 3.2 PLC activities, DAG, and PA content

**Figure 2:**
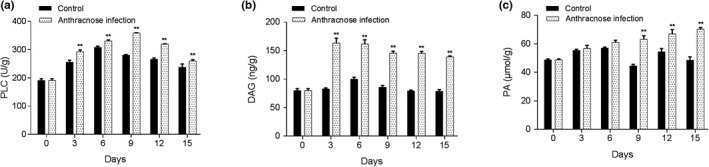
Comparison of the enzyme activities of phospholipase C (PLC) (a), 1,2‐diacylglycerol (DAG) (b) and phosphatidic acid (PA) (c) in the anthracnose infection and control groups. *p < .05 and **p < .01 indicate significant difference with control group

As shown in Figure 2a–c, PLC activity, DAG, and PA content were significantly increased in the banana fruits of the infection group. The results confirmed that the PLC activity of the infected bananas was higher than that of the noninfected bananas. While DAG is derived from the decomposition of membrane components by PLC, the higher the PLC activity, the higher the DAG content. PA can be further decomposed by DAG, where a high PA content indicates stronger resistance ability after anthracnose infection.

### 3.3 Expression levels of MaPLC‐1 and MaPLC‐2

**Figure 3:**
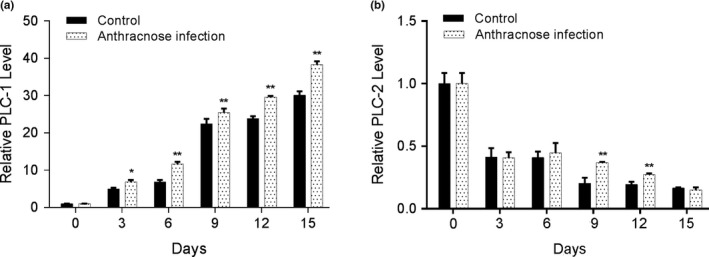
The expression levels of phospholipase C‐1 (*PLC‐1*) (a) and *PLC‐2* (b) in the anthracnose infection and control groups were measured using qPCR. *p < .05 and **p < .01 indicate significant difference with control group

The expression levels of MaPLC‐1 and MaPLC‐2 in the anthracnose infection and control groups were measured using qPCR. Figure 3a showed that the expression levels of MaPLC‐1 were increased in banana samples from the experimental group. The expression levels of MaPLC‐2 in banana samples in the anthracnose infection group were higher than those in the control group on days 9 and 12 (Figure 3b), indicating that MaPLC‐1 played a role in anthracnose infection.

**Figure 4:**
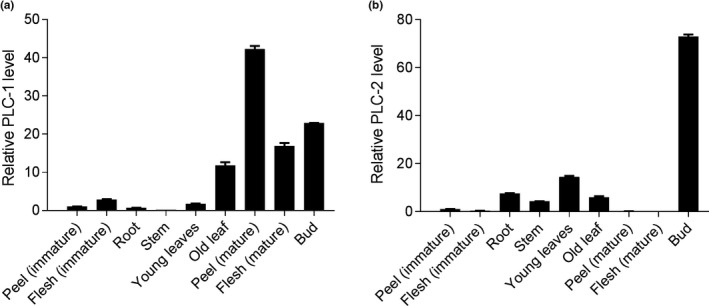
The expression levels of the *PLC‐1* gene (a) and *PLC‐2* gene (b) in the different tissues of banana fruits

The expression levels of *MaPLC‐1* and *MaPLC‐2* among different tissues were also determined. As shown in Figure 4a, the expression levels of *MaPLC‐1* in the immature flesh, young and old leaves, mature peel and flesh, and bud were higher than that in the immature peel; however, compared with the expression levels of *MaPLC‐1* in the immature peel, the expression levels in the root and stem were decreased. We also observed that the mature peel had the highest *MaPLC‐1* expression level, suggesting that *MaPLC‐1* plays important roles in the process of fruit ripening and aging. Notably, the expression levels of *MaPLC‐2* in the root, stem, young leaves, and bud were higher than that in the immature peel. Compared with the expression levels of *MaPLC‐2* in the immature peel, the expression levels in the immature flesh and mature peel and flesh were decreased (Figure 4b). The low expression levels of *MaPLC‐2* gene in the fruit and high expression levels in the flower organs suggest that the expression levels of *MaPLC‐2* are related to fruit development.

## 4 DISCUSSION

Banana is a major commercial fruit crop (Wang et al., 2014; Wang, Luo, Mao, & Ying, 2015), and banana anthracnose is a common postharvest disease of banana fruit caused by *Calletotrichum musae,* which widely occurs in all banana areas. Synthetic fungicides have been the most commonly used treatment option; however, health concerns have increased with societal development (Anthony, Abeywickrama, Dayananda, Wijeratnam, & Arambewela, 2004; Goh & Kasabov, 2005). Therefore, the use of chemical fungicides to treat postharvest diseases of fruit has been controlled (Mahfoudhi, Chouaibi, & Hamdi, 2014). Thus, for the banana industry, discovering sustainable, nonchemical alternative fungicides to control postharvest diseases is an added pressure. Our study tried to evaluate the role of PLC in anthrax resistance in banana.

In our study, the bananas were divided into two groups: the experimental group, which was treated with a banana anthracnose conidia suspension, and the control group, which was treated with distilled water. Then, hardness and conductivity changes; PLC, DAG, and PA activities; and *MaPLC‐1* and *MaPLC‐2* expression levels were assessed. Our data revealed that, compared with the control group, the banana samples in the anthracnose infection group showed lower hardness and higher conductivity. Higher PLC activity, DAG, and PA content were found among samples from the experimental group. Moreover, the mature peel had the highest *MaPLC‐1*expression level, and relatively higher *MaPLC‐1* mRNA expression levels were detected in anthracnose‐inoculated tissues. The low expression levels of the *MaPLC‐2* gene in the fruits and high expression levels in the flower organs suggest that the expression levels of *MaPLC‐2* are related to fruit development.

Traditionally, the quality of export banana fruits was mainly affected by wound anthracnose, which was caused by the pathogenic fungus *Colletotrichum musae* (Ranasinghe, Jayawardena, & Abeywickrama, 2002). In plantations, during the month after flowering, banana fruits would be infected with *Colletotrichum musae* conidia, and melanized appressoria would be germinated. Finally, quiescentanthracnose would be developed by these appressoria when the bananas ripen. Wound anthracnose mainly develops during the hot rainy seasons and primarily affects banana fruits. Bananas in the mountain areas generally seem to be less susceptible to anthracnose due to their different characteristics, such as fruit firmness and ethylene production after peel wounding (Ehrlich & Cotty, 2002; Prusky & Lichter, 2007). Therefore, the characteristics of the banana fruit, such as hardness and electrical conductivity, were detected in our study.

In animals, PLC functions as a major membrane phospholipid‐hydrolyzing enzyme and generates signaling messengers, such as DAG and IP3. In plants, their phosphorylated forms, such as phosphatidic acid and inositol hexakisphosphate, are thought to regulate various cellular processes (Pfaffmann, Hartmann, Brightman, & Morre, 1987; Singh, Bhatnagar, Pandey, & Pandey, 2015). Previous studies have demonstrated that PLC activity is related to various factors, including Ca^2+^ concentration, phospholipid substrate, post‐translational modifications, and interacting proteins (Brearley, Parmar, & Hanke, 1997; Zheng, Krishnamoorthi, Zolkiewski, & Wang, 2000). PLC members are implicated in membrane lipid hydrolysis function, various cellular processes, and signaling networks. Therefore, most researchers have focused on potential candidate exploration for their roles in stress tolerance and crop productivity (Abd‐El‐Haliem & Joosten, 2017; Rupwate & Rajasekharan, 2012). In our study, decreased hardness and increased electrical conductivity of banana fruits were observed when the banana fruit was treated with a banana anthracnose conidia suspension. Anthracnose infection that causes cell membrane destruction and cell wall degradation has been well demonstrated in previous studies (Xu et al., 2017; Zhao, Deng, Zhou, Yao, & Zeng, 2018). The characteristics of fruit hardness and electrical conductivity have been widely used as indexes to evaluate fruit quality (Zhang, 2010). Moreover, these characteristics are all related to cell membrane structure (Wanga, Boussetta, Lebovka, Lefebvre, & Vorobiev, 2018). Therefore, our data might indicate that anthracnose infection may destroy the cell membrane structure of banana fruits.

PLC is an enzyme that hydrolyzes plasma membrane phospholipids at the ester bond of the third position of the glycerol backbone (Fain, 1990). The PLC family comprises multiple members in plants (Canonne, Froidure‐Nicolas, & Rivas, 2011), and PLCs have been reported to play an important role in plant immunity (de Lima Castro et al., 2017; Tsai & Chung, 2014), depending on the types of pathogens and specific PLCs (Legendre et al., 1993; van der Luit et al., 2000). A previous study demonstrated that three phosphoinositide‐specific PLCs were mapped by the anthracnose resistance gene (Richard et al., 2014). In tomato, it was proposed that PI‐PLC is a required factor for hypersensitive response and disease resistance (Vossen et al., 2010). Haliem and colleagues demonstrated an important role of PLC enzymes in plant defense signaling downstream of immune receptors (Abd‐El‐Haliem et al., 2016). Therefore, PLC might also be a key player in anthracnose defense. In our study, bananas inoculated with anthracnose showed higher PLC activity, DAG, and PA content. Increased expression levels of *PLC‐1* were also observed. PLC hydrolyzes membrane phospholipids to generate PA, which is emerging as an important plant lipid second messenger. PA is rapidly and transiently generated in response to different biotic and abiotic stresses via the PLC/DGK pathway (Munnik & Testerink, 2009). DAG is derived from the decomposition of cell membrane components by PLC, which is recognized as a molecular signal in the early stages of anthracnose (Dickman, Buhr, Warwar, Truesdell, & Huang, 1995). Although no study has been proposed regarding the role of PLC in banana anthracnose defense, our data support the idea that PLC‐mediated signaling is involved in banana anthracnose defense.

In conclusion, PLC might play a role in response to banana anthracnose resistance. However, this role should be further verified by constructing gene knockout or overexpression models of the plant.

## CONFLICT OF INTEREST

Author Liang Shuai declares that he has no conflict of interest, Author Li Li declares that she has no conflict of interest. Author Jian Sun declares that he has no conflict of interest. Lingyan Liao declares that she has no conflict of interest. Zhenhua Duan declares that he has no conflict of interest. ChangbaoLi declares that he has no conflict of interest. XuemeiHe declares that she has no conflict of interest.

## ETHICAL APPROVAL

This article does not contain any studies with human participants or animals performed by any of the authors.
